# Supplementary material for: Spreading potential in disease relevant networks: Predicting centralities in rural Northeast Madagascar
Source: PLOS Glob Public Health. 2026 Jan 28;6(1):e0005661. doi: 10.1371/journal.pgph.0005661 (PMC12851470; doi:10.1371/journal.pgph.0005661)
Supplement: S1 Fig — Villages remain unnamed to protect the participants in the small villages (i.e., participants may be identified based on age, sex, and household characteristics due to the small village sizes). The Marojejy National Park shapefile was from UNEP-WCMC and IUCN (2025); the Madagascar country map shapefile was from Natural Earth (Natural Earth Data 2025) using the R package rnaturalearth (Massicotte and South, 2025). (DOCX) [file pgph.0005661.s001.docx]

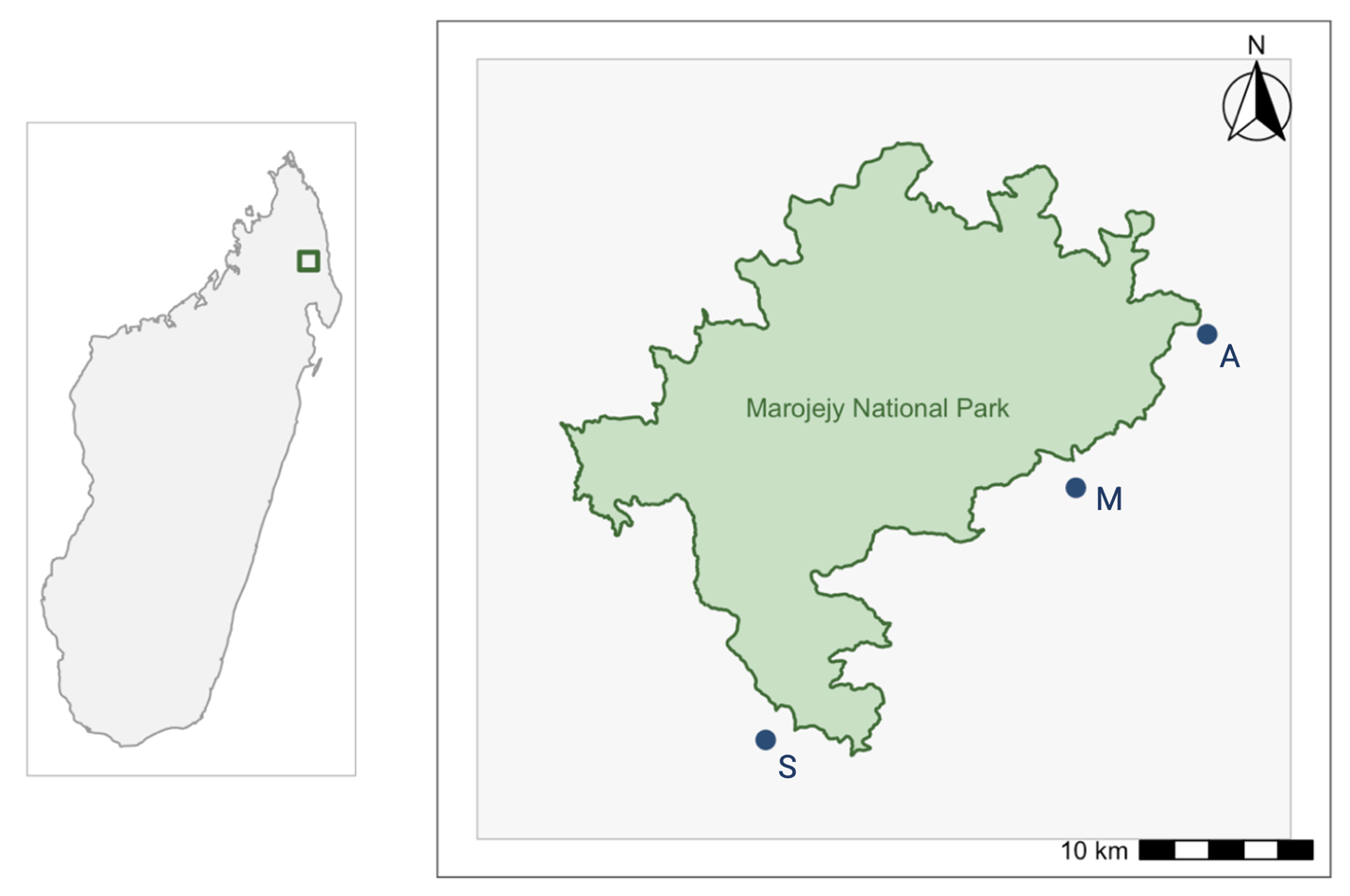


**Supplemental Figure 1**. Map of Madagascar (left) and the three villages covered by the study along the boundary of Marojejy National Park in the SAVA region of northeastern Madagascar (right, three blue dots). Villages remain unnamed to protect the participants in the small villages (i.e., participants may be identified based on age, sex, and household characteristics due to the small village sizes). The Marojejy National Park shapefile was from UNEP-WCMC and IUCN (2025)^1^; the Madagascar country map shapefile was from Natural Earth (Natural Earth Data 2025)^2^ using the R package rnaturalearth (Massicotte and South, 2025)^3^.

**References**

1. UNEP-WCMC and IUCN (2025), Protected Planet: The World Database on Protected Areas (WDPA) and World Database on Other Effective Area-based Conservation Measures (WD-OECM) [Online], December 2025, Cambridge, UK: UNEP-WCMC and IUCN. Available at: www.protectedplanet.net.
2. Natural Earth Data (2025). <https://www.naturalearthdata.com/>
3. Massicotte, P. and South, A. (2025). rnaturalearth: World Map Data from Natural Earth_doi:10.32614/CRAN.package.rnaturalearth <https://doi.org/10.32614/CRAN.package.rnaturalearth>, R package version 1.1.0, <https://CRAN.R-project.org/package=rnaturalearth>
