# Supplementary material for: Spreading potential in disease relevant networks: Predicting centralities in rural Northeast Madagascar
Source: PLOS Glob Public Health. 2026 Jan 28;6(1):e0005661. doi: 10.1371/journal.pgph.0005661 (PMC12851470; doi:10.1371/journal.pgph.0005661)
Supplement: S2 Fig — (b) PCA biplot of the first two principal components, with points representing individuals and arrows representing variable loadings for the six wealth indicators. (c) Pearson correlation heatmap of the six wealth indicators. (DOCX) [file pgph.0005661.s002.docx]

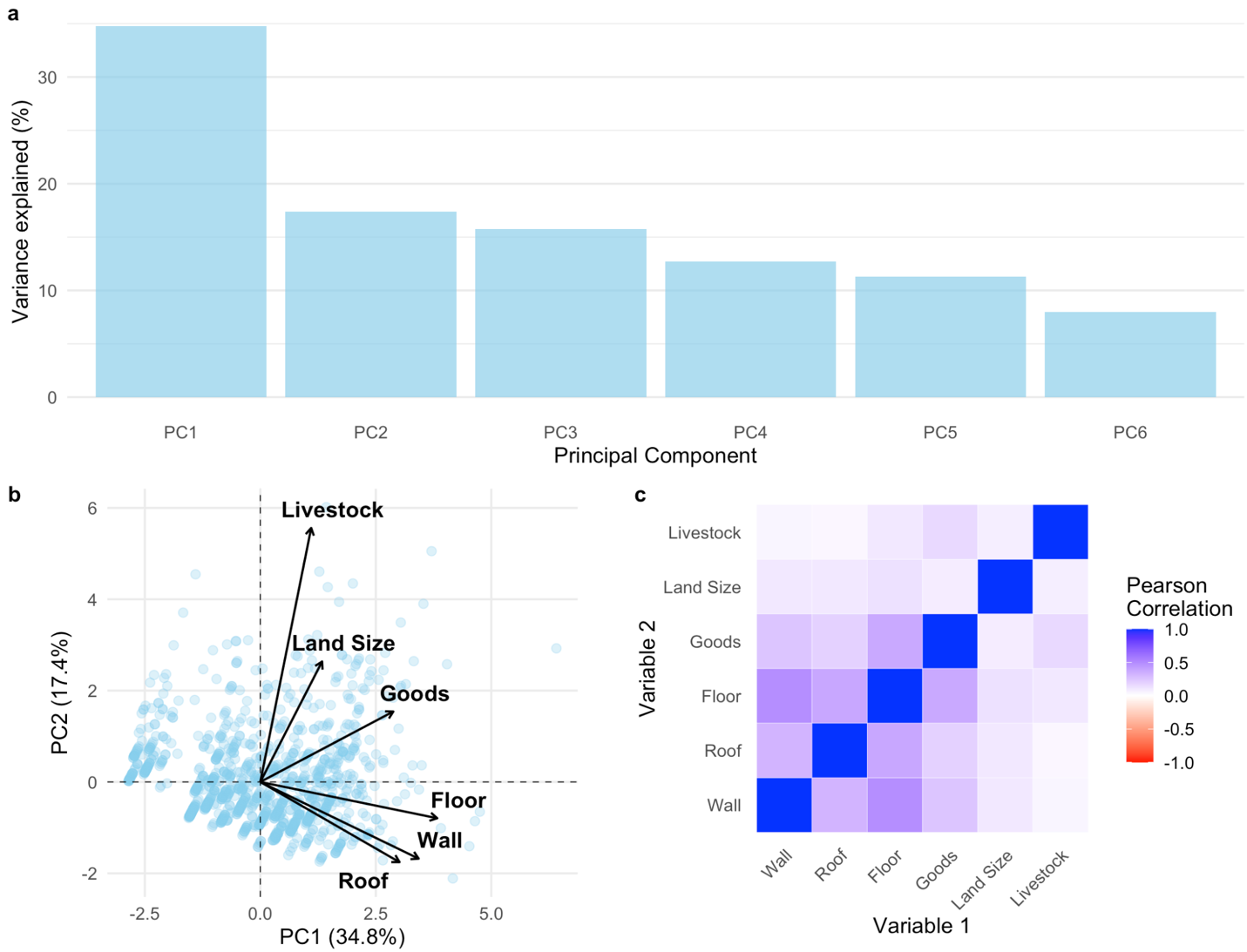

**Supplemental Figure 2**. Results of the principal component analysis (PCA) of wealth indicators: **(a)** Scree plot showing the proportion of variance explained by each principal component. **(b)** PCA biplot of the first two principal components, with points representing individuals and arrows representing variable loadings for the six wealth indicators. **(c)** Pearson correlation heatmap of the six wealth indicators.
