# Supplementary material for: Spreading potential in disease relevant networks: Predicting centralities in rural Northeast Madagascar
Source: PLOS Glob Public Health. 2026 Jan 28;6(1):e0005661. doi: 10.1371/journal.pgph.0005661 (PMC12851470; doi:10.1371/journal.pgph.0005661)
Supplement: S3 Fig — Coefficient plots of the relationships between village, season, and centrality metric for each network type (a. Social Network, b. Close Contact Network, c. Household Network, and d. Environmental Network) when controlling for the sampling date index. Points represent estimated effects; thick bars represent 90% confidence intervals and thin bars represent 95% confidence intervals; color represents variable importance. (DOCX) [file pgph.0005661.s003.docx]

**Supplemental Figure 3**. Sensitivity analysis of including an index for sampling date in the models to account for the snowball sampling method. Coefficient plots of the relationships between village, season, and centrality metric for each network type (**a**. Social Network, **b**. Close Contact Network, **c**. Household Network, and **d**. Environmental Network) when controlling for the sampling date index. Points represent estimated effects; thick bars represent 90% confidence intervals and thin bars represent 95% confidence intervals; color represents variable importance.
