# Supplementary material for: Spreading potential in disease relevant networks: Predicting centralities in rural Northeast Madagascar
Source: PLOS Glob Public Health. 2026 Jan 28;6(1):e0005661. doi: 10.1371/journal.pgph.0005661 (PMC12851470; doi:10.1371/journal.pgph.0005661)
Supplement: S5 Fig — Thick bars represent 95% confidence intervals and thin bars represent 90% confidence intervals. Each individual or household level variable is represented by a distinct color. Point shapes represent the centrality types. (DOCX) [file pgph.0005661.s005.docx]

**Supplemental Figure 5**. Coefficient plots of the relationships between socio-demographic variables and different centrality measures for each network type (**a**. Social Network, **b**. Close Contact Network, **c**. Household Network, and **d**. Environmental Network). Thick bars represent 95% confidence intervals and thin bars represent 90% confidence intervals. Each individual or household level variable is represented by a distinct color. Point shapes represent the centrality types.
