# Supplementary material for: Spreading potential in disease relevant networks: Predicting centralities in rural Northeast Madagascar
Source: PLOS Glob Public Health. 2026 Jan 28;6(1):e0005661. doi: 10.1371/journal.pgph.0005661 (PMC12851470; doi:10.1371/journal.pgph.0005661)
Supplement: S6 Fig — Color represents correlation value. (DOCX) [file pgph.0005661.s006.docx]

**Supplemental Figure 6.** Correlations between centrality scores for the **a.** social, **b.** close contact, **c.** household, and **d.** environmental networks. Color represents correlation value.
