# Supplementary material for: Spreading potential in disease relevant networks: Predicting centralities in rural Northeast Madagascar
Source: PLOS Glob Public Health. 2026 Jan 28;6(1):e0005661. doi: 10.1371/journal.pgph.0005661 (PMC12851470; doi:10.1371/journal.pgph.0005661)
Supplement: S7 Fig — Points represent estimated effects; thick bars represent 90% confidence intervals and thin bars represent 95% confidence intervals; color represents variable importance. The village base factor is Village A; the season base factor is Season 1; the centrality type base factor is betweenness. (DOCX) [file pgph.0005661.s007.docx]

**Supplemental Figure 7**. Coefficient plots of the relationships between village, season, and centrality metric for each network type (**a**. Social Network, **b**. Close Contact Network, **c**. Household Network, and **d**. Environmental Network). Points represent estimated effects; thick bars represent 90% confidence intervals and thin bars represent 95% confidence intervals; color represents variable importance. The village base factor is Village A; the season base factor is Season 1; the centrality type base factor is betweenness.
