# Supplementary material for: Spreading potential in disease relevant networks: Predicting centralities in rural Northeast Madagascar
Source: PLOS Glob Public Health. 2026 Jan 28;6(1):e0005661. doi: 10.1371/journal.pgph.0005661 (PMC12851470; doi:10.1371/journal.pgph.0005661)
Supplement: S2 Table — This table contains the full model results and numeric outputs of the evaluated and plotted predictors in Fig 2. (DOCX) [file pgph.0005661.s009.docx]

| **Variable**  **Type** | **Variable** | **Social** | | | **Close Contact** | | | **Household** | | | **Environmental** | | |
| --- | --- | --- | --- | --- | --- | --- | --- | --- | --- | --- | --- | --- | --- |
|  |  | **Est.** | **Std. Error** | **Impt.** | **Est.** | **Std. Error** | **Impt.** | **Est.** | **Std. Error** | **Impt.** | **Est.** | **Std. Error** | **Impt.** |
| Intercept | | -0.093 | 0.058 | NA | 0.077 | 0.050 | NA | 0.065 | 0.038 | NA | 0.047 | 0.079 | NA |
| Socio- demographic | Gender [Man] | 0.056 | 0.064 | 0.507 | -0.017 | 0.039 | 0.196 | -0.001 | 0.009 | 0.041 | 0.387 | 0.059 | 1 |
|  | House Material | 0.000 | 0.003 | 0.02 | 0.083 | 0.029 | 0.956 | 0.002 | 0.008 | 0.056 | 0.020 | 0.035 | 0.286 |
|  | Goods | 0.001 | 0.006 | 0.035 | -0.002 | 0.010 | 0.057 | 0.004 | 0.012 | 0.1 | 0.017 | 0.033 | 0.256 |
|  | Household Size | -0.001 | 0.005 | 0.031 | 0 | 0.004 | 0.026 | 0.001 | 0.006 | 0.037 | 0.001 | 0.006 | 0.033 |
|  | Livestock | 0.020 | 0.030 | 0.362 | 0 | 0.004 | 0.022 | 0 | 0.004 | 0.023 | 0.001 | 0.006 | 0.031 |
|  | BMI | 0.001 | 0.007 | 0.042 | 0 | 0.005 | 0.027 | 0 | 0.003 | 0.02 | -0.002 | 0.011 | 0.053 |
|  | Education | 0.001 | 0.007 | 0.043 | 0 | 0.005 | 0.026 | 0 | 0.003 | 0.018 | -0.005 | 0.019 | 0.103 |
|  | Land Size | -0.001 | 0.007 | 0.039 | 0.003 | 0.011 | 0.073 | 0.003 | 0.012 | 0.096 | 0.010 | 0.025 | 0.162 |
|  | Age | 0.001 | 0.006 | 0.037 | 0.019 | 0.030 | 0.322 | 0.003 | 0.012 | 0.097 | 0.005 | 0.018 | 0.098 |
| Control | Village [M] | 0.476 | 0.06 | 1 | 0.265 | 0.074 | 0.998 | 0.217 | 0.05 | 1 | 0.227 | 0.139 | 0.783 |
|  | Village [S] | 0.35 | 0.05 | 1 | 0.197 | 0.053 | 0.998 | 0.452 | 0.042 | 1 | 0.035 | 0.063 | 0.783 |
|  | Centrality [Eigenvector] | 0 | 0 | 0 | 0 | 0 | 0 | 0 | 0 | 0 | 0 | 0 | 0 |
|  | Centrality [PageRank] | 0 | 0 | 0 | 0 | 0 | 0 | 0 | 0 | 0 | 0 | 0 | 0 |
|  | Centrality [Strength] | 0 | 0 | 0 | 0 | 0 | 0 | 0 | 0 | 0 | 0 | 0 | 0 |
|  | Season 2 | -0.240 | 0.053 | 0.997 | -0.160 | 0.061 | 1 | -0.184 | 0.044 | 1 | -0.275 | 0.071 | 1 |
|  | Season 3 | -0.239 | 0.060 | 0.997 | -0.536 | 0.067 | 1 | -0.698 | 0.050 | 1 | -0.558 | 0.089 | 1 |
