# Supplementary material for: Spreading potential in disease relevant networks: Predicting centralities in rural Northeast Madagascar
Source: PLOS Glob Public Health. 2026 Jan 28;6(1):e0005661. doi: 10.1371/journal.pgph.0005661 (PMC12851470; doi:10.1371/journal.pgph.0005661)
Supplement: S1 Text — (DOCX) [file pgph.0005661.s010.docx]

**Spreading Potential in Disease Relevant Networks: Predicting Centralities in Rural Northeast Madagascar**

Supplemental Material

*Principal Components Analysis (PCA)*

To analyze how the centrality predictors related and to reduce the dimensionality of our wealth data, we conducted a principal components analysis (PCA). The variance in wealth was distributed across multiple components and a single axis explained most of the variation. Since our wealth data were multidimensional, we analyzed distinct dimensions of wealth separately. We also avoided categorizing households into wealth quintiles to retain the continuous nature of the underlying variables, thereby preserving the ability to detect fine-scale variation in wealth among individuals.

Methods: To create a composite index of wealth that captures variation in the indicators used in our analysis, we conducted a principal component analysis (PCA). We used the six wealth variables included in our analysis: house wall material, house roof material, and house floor material (scored according to the process described in the main text), commercial goods ownership, land size, and livestock owned. Commercial goods ownership was a sum of binary responses for the ownership of seven goods: cell phone, television, bicycle, refrigerator, motorcycle, computer, and generator. All six variables were normalized prior to analysis. We conducted a PCA to reduce these correlated indicators into composite wealth measures. We then examined PCA scree plot, PCA biplot, and pairwise variable correlations.

Results & Interpretation: The first principal component (PC1) explained 34.8% of the variation in the data, followed by PC2 which accounted for 17.4% of variation (**Supplemental Figure 2a**). Four principal components are necessary to explain a cumulative 80% of the variation in the data. This distribution indicates that wealth is multidimensional and that people differ across multiple different axes of wealth. PC1 had positive loadings for all six variables, indicating that it represents a general wealth gradient (**Supplemental Figure 2b**). PC2 was positively associated with the number of livestock owned, land size, and the number of commercial goods owned, but negatively associated with house materials. Additionally, while all wealth variables were positively correlated, they were only weakly correlated (**Supplemental Figure 2c**). Taken together, the multidimensional nature of the wealth data and weak correlation among wealth variables suggest that analyzing multiple dimensions of wealth separately is the approach best suited for our analysis. However, all three house material variables loaded similarly onto the first two PCs, providing a justification for combining them into one house materials index. We therefore use the following wealth variables in our analysis: house materials, commercial goods owned, livestock owned, and land size.
