# Supplementary material for: Spreading potential in disease relevant networks: Predicting centralities in rural Northeast Madagascar
Source: PLOS Glob Public Health. 2026 Jan 28;6(1):e0005661. doi: 10.1371/journal.pgph.0005661 (PMC12851470; doi:10.1371/journal.pgph.0005661)
Supplement: Attachment — Submitted filename: PLOS PGH Response 9 23 25.pdf [file pgph.0005661.s013.pdf]

We thank the reviewers and editorial team for their thoughtful and constructive feedback on our manuscript, “Spreading Potential in Disease Relevant Networks: Predicting Centralities in Rural Northeast Madagascar” (PGPH-D-25-01742). Their comments have significantly strengthened the clarity, rigor, and public health relevance of our work. Several common themes emerged across the reviews, including the need for greater detail in the description of methods, additional transparency around data collection and sampling, clearer interpretation of results, and expanded discussion of public health applications.

In response, we have substantially revised the manuscript. Please see below the integrated responses in blue. Note, the line numbers correspond to the manuscript in track changes. In summary, we expanded the Methods section to provide more information on sampling strategies, survey design, GPS data collection procedures, and village-specific population characteristics. We added a map and supplemental materials to give readers clearer context on the study setting, and we incorporated a principal components analysis to strengthen the treatment of socioeconomic indices. We clarified the interpretation of predictors and importance scores, explicitly noting cases of wide confidence intervals and overlapping estimates, and refined the Abstract to present key findings more specifically across network types. Finally, we expanded the Discussion to address potential biases, explain village- and season-specific effects, and highlight how network predictors may inform targeted public health interventions.

We are grateful for the reviewers’ insights, which have guided substantial improvements to both the scientific robustness and the practical significance of the paper.

Journal Requirements:

1. Please include a complete copy of PLOS’ questionnaire on inclusivity in global research in your revised manuscript. We submitted a completed version of the questionnaire as Supporting Information and referenced it in the main text.

2. Please provide separate figure files in .tif or .eps format. We provided the files in the revised submission.

3. **[**Our data contains GPS coordinates of the study participants that cannot be made fully available.**]**

We note that you have indicated that there are restrictions to data sharing for this study. For studies involving human research participant data or other sensitive data, we encourage authors to share de-identified or anonymized data. However, when data cannot be publicly shared for ethical reasons, we allow authors to make their data sets available upon request. For information on unacceptable data access restrictions, please see http://journals.plos.org/plosone/s/data-availability#loc-unacceptable-data-access-restrictions.

Before we proceed with your manuscript, please address the following prompts:

a) If there are ethical or legal restrictions on sharing a de-identified data set, please explain them in detail (e.g., data contain potentially identifying or sensitive patient information, data are owned by a third-party organization, etc.) and who has imposed them (e.g., a Research Ethics

Committee or Institutional Review Board, etc.). Please also provide contact information for a data access committee, ethics committee, or other institutional body to which data requests may be sent.

We are unable to share even a minimal anonymized data set because a clause in the consent form stated that individual answers would not be shared publicly, which has been interpreted by the IRB as not sharing any individual level survey data. Likewise, GPS data, which cannot be anonymized, cannot be shared. Hence, to protect the participants and comply with the consent we are unable to share access to the database used for this manuscript.

b) If there are no restrictions, please upload the minimal anonymized data set necessary to replicate your study findings to a stable, public repository and provide us with the relevant URLs, DOIs, or accession numbers. Please see http://www.bmj.com/content/340/bmj.c181.long for guidelines on how to de-identify and prepare clinical data for publication. For a list of recommended repositories, please see https://journals.plos.org/plosone/s/recommended-repositories. You also have the option of uploading the data as Supporting Information files, but we would recommend depositing data directly to a data repository if possible.

Please update your Data Availability statement in the submission form accordingly.
We updated the statement in the portal accordingly.

Reviewer #1:

This study has interesting public health applications when considering disease prevention activities, especially in a rural population.

Many of my comments request clarifying details to the study methods.

The methods section begins with details on data collection. I think the paper would benefit from additional details on the sampling strategy and the survey instrument. For example, how were individuals 18+ selected to be in the study? What proportion of the overall population does the study sample represent?
We added several paragraphs of additional information on sample collection in the Methods to help clarify the setting and sampling strategy, see lines 188-273. Specifically, adult study participants were enrolled through a snowball sampling method, starting with a small group of landowners and their households. Other adult individuals in the community became eligible for enrollment when they were named as someone with whom enrolled individuals spend their free time, exchange farmwork, or exchange food. There is no formal population census data available for our catchment area, so we cannot estimate the exact proportion of the overall population our study covered. However, based on field observations related to estimated community size (estimated population sizes of the villages A, S, and M were 1900, 900, and, 2700 respectively), and the range of age, gender, and socioeconomic status of the participants, we believe that the study captured a sizable portion of the population, and our study cohort is representative of the three villages.

Additionally, details on data collection timing would be helpful. It's noted that data collection happened over a nearly three-year period, but how much of that was active data collection time?

These details would be especially useful for movement data collected with GPS trackers. It's unclear for how long study participants recorded their movement, and how much data collection time varied across study participants and villages.

We added additional information on the sample collection period in the methods, see lines 232-235. Each village was surveyed over multiple seasons (for 3-6 weeks each season). Villages S and A were surveyed in all three seasons; Village M was surveyed in Season 1 and Season 2. We were unable to survey Village M in Season 3 due to the COVID-19 pandemic. The participants could choose to wear a GPS tracker one week at a time and the loggers recorded GPS location every 3 minutes. After each week, fully charged devices replaced the previous one. The movement of one individual was tracked for up to 6 weeks during each season. The participants were asked to wear the trackers at all times during the day.

We note in the Discussion that, based on this study design, there is potential for selection bias, see lines 659-667. Due to the snowball sampling, participants who were enrolled later may have been inherently less likely to be well connected in the networks. Additionally, season-dependent weather could have driven differential movement patterns. Also, varying village-dependent environmental factors and sizes could have led to different social patterns, movements, and household layouts. We attempted to minimize the potential selection biases by controlling for village and sampling season in the models and by attempting to maximize the proportion of participants in each village.

I think the reader would also benefit from more details on the survey. Was this part of a larger survey? Are there additional data that could be considered to determine network centrality (e.g. occupation)?

Thank you for the opportunity to clarify the overall context of the study and the associated survey. The analyzed data was indeed collected as part of a larger study (see references about previous study by our team in the region). For this analysis, we aimed to use the collected sociodemographic and anthropometric variables to inform public health intervention strategies. We therefore included the relevant survey data points such as gender, age, education, owned goods, household size, house material index, land size, livestock, and BMI.

The survey collected data on durable goods ownership and household material construction, both of which are included in the model as a continuous index. Often with such scales, a score is constructed using principal components analysis and then individuals or households are broken into quintiles. I wonder if this would have an impact on results or be a more useful framing for understanding how wealth and centrality relate.

We agree that it is valuable to consider wealth dimensionality reduction. We have added a principal components analysis (PCA) and the respective results in the main text, see lines 295-302, and in the supplemental methods (Supplemental Figure 2). The results from this additional analysis showed that the variance in wealth was distributed across multiple components and a single axis explained most of the variation. Since our wealth data were multidimensional, we decided to analyze distinct dimensions of wealth separately. We also avoided categorizing households into wealth quintiles, instead retaining the continuous nature of the underlying variables to better analyze fine-scale variation in wealth among individuals.

In Table 1, the BMI distribution and percentage of people attending higher education represent a higher proportion of the study sample than I would have expected. Additional information in the

methods on how the sample was collected and some background information on the study area would help provide context for the reader on how representative this sample is. Also, a map to indicate how geographically proximate the three villages are could be helpful.
We added a new section in the methods that now covers more information about the villages and population we sampled, see lines 188-273. We also added a map with the villages, see Supplemental Figure 1. However, to protect the study participants, we did not label the villages. Given the small village sizes, participants may be identifiable based on age, sex, household characteristics, and/or GPS-associated data points.

The discussion could include more reflection on how different network types and demographic predictors of centrality could be used to inform public health interventions.
Thank you for this helpful suggestion. We added additional text and references in the Discussion covering how different network types and the respective measured predictors relate to public interventions, see lines 550-584. In summary, the study examines four types of networks that capture different pathways of infectious disease transmission. Social networks reflect long-term relationships and shared resources, which can guide education-based interventions. GPS-based close contact networks capture direct physical proximity, which can help to identify high-risk interactions. Household networks represent within-village contact through shared living environments, while environmental networks capture overlapping land use outside villages. Together, these networks provide complementary insights into transmission risks inside and outside villages and have the potential to inform targeted public health interventions.

Reviewer #2:

This study investigates how sociodemographic and anthropometric characteristics predict individuals' centrality in different contact networks. Incorporating GPS tracking and survey data in a large cohort of participant, the authors employed mixed-effect linear models and found gender and household material index (a proxy for wealth) to be often associated with higher centrality across networks.

This study addresses an important topic in network epidemiology: identifying likely "superspreaders" via low-cost but effective predictors. While the paper has potential, several methodology and interpretation concerns need to be addressed:

Major concerns:

1. While the study enrolled a large number of participants (n=1220), I was not able to gauge what proportion of the entire population was involved in this study without further information. The primary concern lies in the fact that the close contact and household networks tracked only participants in this study, but had no imputation or other means to account for those who did not participate. How is selection bias being addressed in the models?
We agree that it is important to include this information to increase the transparency of our methods and interpretability of our results. Accordingly, we added a new section in the Methods that now covers more information on the villages and population we sampled, see lines 188-273. Adult study participants were enrolled through a snowball sampling method, starting with a small group of landowners and their households. Other adult individuals in the community became

eligible for enrollment when they were named as someone with whom enrolled individuals spend their free time, exchange farmwork, or exchange food. There is no formal population census data available for our catchment area, so we cannot estimate the exact proportion of the overall population our study covered. However, based on field observations related to estimated community size (estimated population sizes of the villages A, S, and M were 1900, 900, and, 2700 respectively), and the range of age, gender, and socioeconomic status of the participants, we believe that the study captured a sizable portion of the population, and our study cohort is representative of the three villages.

We also added a map with the villages to give more information on the geographic setting, see Supplemental Figure 1. To protect the study participants, we did not label the villages.

We now also note in the Discussion that based on this study design, there is potential for selection bias, see lines 659-667. Due to the snowball sampling, participants who were enrolled later may have been inherently less likely to be well connected in the networks and the season-dependent weather could have driven differential movement patterns. Further, varying village-dependent environmental factors and sizes could have led to different social patterns, movements, and household layouts. We attempted to minimize the potential selection biases by controlling for village and sampling season in the models and by attempting to maximize the proportion of participants in each village.

2. Related to the first concern, was any incentive paid to the participants for them to wear the GPS tracker? What were the exact instructions provided to the participants about wearing the GPS device (e.g., wear it at all times vs. feel free to remove it as needed)?
We added a new section in the Methods that now covers more information on the GPS data collection, see lines 305-330. Participants were compensated with pre-paid cell phone cards worth about $USD 0.75 for each week they wore the device. The participants were asked to wear the trackers at all times during the day, and the present analysis was limited to daytime hours to capture the active day period.

3. Most of the predictors were not statistically significant in this study. For example, gender was the most important centrality predictor for the social network, but its confidence interval was wide and included 0. Does this suggest that even though gender and livestock ownership were important predictors, we cannot say much about the direction of their effects on centrality at a 95% confidence level? In general, I would be very careful about interpreting the results when the CI is wide and overlaps 0.
Thank you for the comment. Yes, we tried to clarify that CIs were wide/overlap with zero and that the interpretation is also based on the overall importance value. For example, we mention in the manuscript that "the confidence intervals of the effects of both gender and livestock ownership on social network centrality overlapped zero" (lines 486-502). In the Discussion we state that "gender and wealth were the strongest predictors of centrality in agricultural communities of rural Northeast Madagascar, but the effect of these predictors was often uncertain, low, and variable across network types. Still, with these and other proxies, it is possible to rank people according to their likely centralities. Interventions may therefore consider using these data to monitor, treat, or vaccinate those with highest observed or predicted centrality to maximize the impact of intervention effort." (lines 668-673). As for the gender and livestock ownership - yes, based on our results those predictors tend to be included in the best models for predicting centrality and they cross zero in some networks. However, male gender is clearly positive for the environmental and

social networks and does not cross zero for the environmental network. Hence, the direction is clear in that case.

4. For the environmental network, gender had an importance score of 1. What does this mean, and how should I interpret this result statistically?
Thank you for pointing out that the importance score needs more explanation. Importance is the sum of AIC weights for all models in the candidate set that included that variable. For example, the importance score of 1 means that, across our model set of all candidate models used in model averaging, the gender variable was included in all models. Hence, importance represents the probability that a given variable is included in the best model, out of the comprehensive suite of models used in the model averaging. We now included this explanation in the Methods as well, see lines 399-414.

5. There is an imbalance in terms of household size and land size among the three villages. In particular, Village M had the highest proportion of households with more than 5 members, but the lowest proportion with large land sizes, which suggests a higher density of residents in this area. Does population density and/or other differences between the villages play a role in this analysis?
We have added more information on the village settings, see lines 188-273. It is true that Village M is the biggest of the three and likely most dense. However, there should be no effect on the present analysis, because we control for village-specific effects in the model. Still, future work with more villages (n=3 is limiting) and more population ranges are needed to study changes in transmission potential patterns depending on population density and other village-specific factors.

Minor concerns & typos:

1. The Results & Conclusions in the Abstract are too generic. I would recommend spelling out the key findings for each network type.
We have adjusted and expanded the Results & Conclusions in the Abstract.

2. The authors noted that “village and sampling season tended to be the strongest predictors of centrality across network types” without providing more details about why this would have been the case. I suggest that the authors elaborate further and explain, for example, how seasonality affects the centrality in the analysis?
The village selection and the seasonal spread of the sampling may have led to selection biases and may have been strong predictors because (i) season-dependent weather could have driven differential movement patterns and (ii) village-dependent environmental factors and sizes could have led to different social patterns, movements, and household layouts. We attempted to minimize this selection bias by controlling for village and sampling season in the model and maximizing the participant number (i.e., overall representation), see description in Discussion, lines 659-667.

3. I found Supplementary Table 1 to be pretty helpful, in addition to the visualization in Figure 2. I suggest that the authors clean up and summarize the model results (with importance added as another column) and report the numbers.
Thank you for that suggestion. The table is now cleaned up and expanded, including the associated importance values, see Supplemental Table 2. This was originally Supplemental Table 1 but the numbering was adjusted to streamline the numbering and start with #1.

4. On Page 13 Line 250, a decimal point was mistyped as a comma.
Thank you for catching the typo. It is now corrected.
