# Supplementary material for: Spreading potential in disease relevant networks: Predicting centralities in rural Northeast Madagascar
Source: PLOS Glob Public Health. 2026 Jan 28;6(1):e0005661. doi: 10.1371/journal.pgph.0005661 (PMC12851470; doi:10.1371/journal.pgph.0005661)
Supplement: Attachment — Submitted filename: PLOS PGH Response_Dec 1 2025 .pdf [file pgph.0005661.s014.pdf]

We thank the reviewers and editorial team for the additional feedback on our manuscript, "Spreading Potential in Disease Relevant Networks: Predicting Centralities in Rural Northeast Madagascar" (PGPH-D-25-01742R1). The feedback has significantly improved the public health framing and clarity of the applied methods. We agreed with the need for clearer pathogen-specific relevance, more discussion around the sampling biases from the snowball recruitment method, additional details on the network construction approach, and a more cautious interpretation of the observed effects.

In response, we have substantially revised the manuscript. Please see below the integrated responses in blue. Note, the line numbers correspond to the manuscript in track changes. In summary, we improved the Methods section to provide more information on sampling strategies and network methodology. We added a sensitivity analysis to the supplemental materials to give readers more information on potential biases introduced by the snowball sampling and incomplete population coverage. We clarified the choices and interpretation of centrality metrics in epidemiological and pathogen-specific terms and refined the Abstract. Finally, we expanded the Discussion to address potential biases better, expand on village- and season-specific effects, and highlight how network predictors may inform pathogen-specific public health interventions. Note, we also added a funding source we had previously missed.

We are grateful for the reviewers' insights, which have guided substantial improvements to both the scientific robustness and the practical significance of the paper.

Reviewer #2: All my comments have been addressed in the revision. The authors' approach to address and minimize selection bias inherent in the sample mechanism is reasonable, and the study results can add great value to the existing literature. I enjoyed reading the revised manuscript. Great work!

We greatly appreciated the original feedback, which we found highly constructive. Thanks again.

Reviewer #3:
This manuscript explores how sociodemographic and anthropometric factors predict network centralities in four types of "transmission-potential" networks (social, close contact, household, and environmental overlap) in rural northeast Madagascar. The authors integrate survey and GPS-based data with network and mixed-effects modeling approaches to identify predictors of centrality that may correspond to higher disease transmission potential.

The study is methodologically ambitious. The integration of social, spatial, and epidemiological perspectives is commendable, and the data collection appears rigorous and ethically conducted. However, while the work is innovative, it requires stronger contextualization, a clearer epidemiological framing, and more cautious interpretation of statistically weak effects. Several methodological clarifications are also necessary before the manuscript can be accepted.

Major Comments

1. Epidemiological Relevance and Framing. The manuscript discusses "transmission-potential networks" abstractly but does not link the findings to specific pathogens or transmission modes relevant to Madagascar. Discussing about which pathogens could be relevant in this context, especially according to their transmission routes, is important to add.

We agree that strengthening our epidemiological framing will improve the manuscript. We added information on specific pathogens and transmission modes in the Discussion on lines 1265-1271 and in the Abstract. Based on our results, infections acquired through shared soil or water sources outside of household settings - such as hookworm or *Giardia* - should primarily focus on men. In contrast, when pathogens are spread through close interpersonal contact within homes or village environments, particularly via fecal–oral routes - such as norovirus or *Shigella* - women may be the more appropriate target for interventions.

2. Sampling and Representativeness. The snowball sampling approach is understandable for social network analysis, yet it introduces strong structural biases (especially under-representation of peripheral nodes). The authors should discuss more explicitly how this design, maybe through simulations, may affect the observed centrality patterns and whether the resulting networks approximate whole-community structures.

Thank you for the opportunity to clarify our sampling approach and how it may induce bias into our results. We have carefully considered the role of bias in our analysis. We added a sensitivity analysis, which showed that estimates for individual- and household-level predictors were stable across sampling dates. These results are provided in Supplemental Figures 6 and 7. Prior work also indicates that, for networks similar to ours, even >70% missing data, biased toward peripheral nodes, maintain high correlation (≈0.9) for degree measures. Thus, substantial and highly biased missingness would be required to meaningfully alter/bias results. We noted this in the Methods, and we added a new paragraph in the Discussion section, lines 1336-1402, reviewing potential selection bias in our study.

3. Network Construction Details. While Table 1 summarizes the types of networks, key methodological details remain vague—particularly the criteria for edge weighting and the treatment of non-synchronous GPS tracking. A clear schematic or expanded supplementary table explaining how proximity thresholds and environmental overlaps were defined would strengthen reproducibility.

Thank you for this useful suggestion. Table 1, which contains information on Network measurement approaches, now includes an additional column detailing the edge weighting methods. We also added more details on the applied methodology, see lines 686-733 in Methods. Note, more details on the exact network methods used for this publication can be found in the Kauffman et al. publication as well (reference number 14).

4. Choice and Interpretation of Centrality Measures. The paper uses multiple centrality metrics (strength, PageRank, eigenvector, betweenness) without fully explaining their epidemiological meaning. Why, for instance, is PageRank used for undirected networks? A short conceptual justification of each metric's relevance to transmission would help readers interpret the results.

In addition to the description in other sections, we now include more information on the centrality measures and their epidemiological implications in the Discussion section, see lines 1181-1221. Briefly here: We calculated four different centrality metrics because each of them captures slightly different aspects of node connections and therefore of potential infectious disease transmission. Strength accounts for frequency and duration of contact (i.e., weighted version of degree centrality). For close contact infectious disease transmission, it is important to account for contact intensity since

longer contact increases the risk of transmission. PageRank centrality mirrors possible movement of a pathogen or information through a network and is therefore a useful centrality measure when investigating disease transmission. Note, while our networks were undirected, infectious diseases can be spread in single directions. Eigenvector reflects the degree to which a node is connected to other highly connected nodes, and thus also captures connectivity of nodes, making it well suited for studies of infectious disease transmission. Betweenness centrality quantifies the importance of a focal node by calculating the number of shortest paths between all other pairs of nodes that pass through the focal node. Thus, a node with high betweenness centrality is better at connecting other nodes in the network, making betweenness relevant to studies of infectious disease transmission. In our population and networks, the different centrality metrics were positively correlated, while betweenness centrality was the least correlated compared to the other centrality metrics.

5. Village- and Season-Level Variation. Village and season effects appear important, yet the discussion remains brief. Since these represent key ecological and social contexts, the authors should explore (even qualitatively) how these factors might shape contact patterns and centralities differently across locations.

Thank you for pointing out that gap. We added a paragraph on village and season effects to the Discussion, see lines 1300-1315. Individuals sampled in the wet seasons tended to have lower centrality than individuals sampled in the dry season (Supplemental Figure 5). Weather could have driven differential movement patterns, especially for the GPS-based close contact and environmental networks. However, the significant effects of sampling season in the temporally static household network (Supplemental Figure 5c) point towards this effect being at least partially an artifact of snowball sampling. Also, varying village-dependent environmental factors and sizes could have led to different social patterns, movements, and household layouts. For example, individuals in Village A tended to be less central to their networks than individuals in Village M or S. This may reflect that Village A is composed of two proximate communities rather than one cohesive community (see Methods). We advocate for future research across more villages, which would enable quantitative analysis of village level heterogeneities in human contact.

Minor Comments

1. The abstract could be streamlined for clarity and consistency in style (avoid redundancy such as “few individuals are responsible for substantially more secondary infections…”).

We have edited the Abstract accordingly.

2. Figures are informative but could benefit from clearer legends and consistent font size.

We have streamlined the Figure formatting and legends for clarity.

3. A concise summary table highlighting significant predictors by network type would help readers synthesize the results.

To help synthesize the results, we have edited the text throughout (see track changes). We think this makes our results clearer: only gender (being a man) and wealth (in terms of house material

index) turned out to be significant predictors. Men were more central to their environmental overlap networks and participants who lived in houses made of higher quality materials tended to be more central to their close contact networks.
